# Does the Injection Site Matter During CPR? A Systematic Review and Meta-Analysis of Drug Pharmacokinetics and Pharmacodynamics

**DOI:** 10.3390/jcm14217497

**Published:** 2025-10-23

**Authors:** Sofia-Chrysovalantou Zagalioti, Sofia Gkarmiri, Efstratios Karagiannidis, Panagiotis Stachteas, Aikaterini Zgouridou, Panagiotis Zagaliotis, Katerina Kotzampassi, Vasileios Grosomanidis, Nikolaos Raikos, Maria Aggou, Nikolaos Fragakis, Barbara Fyntanidou

**Affiliations:** 1 Department of Emergency Medicine, AHEPA University General Hospital, Aristotle University of Thessaloniki, 541 24 Thessaloniki, Greece; sofia.gkarmiri@gmail.com (S.G.); zgouridou@auth.gr (A.Z.); bfyntan@yahoo.com (B.F.); 2 Second Department of Cardiology, Hippokration General Hospital, Aristotle University of Thessaloniki, 541 24 Thessaloniki, Greecenfrag@auth.gr (N.F.); 3 Transplantation/Oncology Program, Division of Infectious Diseases, Weill Cornell Medicine, New York, NY 10065, USA; panagiotis_zag@yahoo.com; 4 Department of Surgery, Aristotle University of Thessaloniki, 541 24 Thessaloniki, Greece; kakothe@yahoo.com; 5 Department of Anesthesiology and ICU, Faculty of Medicine, Aristotle University of Thessaloniki, 541 24 Thessaloniki, Greece; grosoman@otenet.gr; 6 Laboratory of Forensic Medicine and Toxicology (FT, NR), Medical School, Aristotle University of Thessaloniki, 541 24 Thessaloniki, Greece; raikos@auth.gr; 7 Registered Nurse, AHEPA University Hospital of Thessaloniki, 546 36 Thessaloniki, Greece; maria_aggou@yahoo.gr

**Keywords:** injection site, cardiopulmonary resuscitation, resuscitation drugs, pharmacokinetics, pharmacodynamics, meta-analysis, cardiac arrest

## Abstract

**Background:** Cardiac arrest is a time-critical medical emergency during which prompt and effective drug delivery plays a key role in patient outcomes. Current resuscitation guidelines recommend intravenous (IV) access as the first-line route, with intraosseous (IO) access recommended as an alternative when IV access is delayed or not feasible. Although the endotracheal (ET) route was previously included in resuscitation protocols, it is no longer recommended. This study aims to evaluate the pharmacokinetic (PK) and pharmacodynamic (PD) effects of resuscitation drugs administered through different injection sites and under varying hemodynamic conditions in in vivo animal models. **Methods:** PubMed, CENTRAL and ClinicalTrials.gov were searched up to August 2025 for studies comparing different injection sites for the same drug (adrenaline/epinephrine, amiodarone, lidocaine and vasopressin) during CPR. Study selection, data extraction, and quality assessments were performed independently by two reviewers. Frequentist random-effects models were used to calculate mean differences and odds ratios (ORs) with 95% confidence intervals (CIs). **Results:** Fourteen prospective experimental studies (sample sizes ranging from 15 to 49 animals) conducted on swine were included. For epinephrine under normovolemia, humeral IO (HIO) access achieved significantly higher maximum concentrations (C_max_; *p* = 0.0238) and a shorter time to the maximum concentration (T_max_; *p* < 0.01) compared to IV, translating into faster return of spontaneous circulation (ROSC) (*p* = 0.0681). Under hypovolemia, IV access proved superiority over IO for epinephrine administration (MD = +382.80 ng/mL; *p* = 0.0022). The time to ROSC was significantly shorter with sternal IO (SIO) compared to tibial IO (TIO) (*p* = 0.0109). For amiodarone and vasopressin, no consistent or statistically significant differences were observed between administration routes, and in several cases, the findings were based on a single study. **Conclusions**: The injection site significantly influences the PK and PD of epinephrine during cardiac arrest. Proximal IO routes may offer advantages under normovolemic conditions, while IV access appears superior in cases of hypovolemic shock. Further research is needed to guide optimal drug delivery in varying hemodynamic conditions during cardiac arrest.

## 1. Introduction

Cardiac arrest constitutes a major global public health challenge and one of the most time-critical conditions in medicine [1,2]. In the United States, it is estimated that more than 350,000 out-of-hospital cardiac arrests (OHCAs) and approximately 290,000 in-hospital cardiac arrests (IHCAs) occur annually [3,4]. Despite advances in resuscitation, OHCA continues to be associated with low survival rates, even among patients who achieve return of spontaneous circulation (ROSC) and are admitted to the hospital [5,6]. In contrast, IHCA outcomes have gradually improved over recent years, with nearly one-third of survivors achieving five-year survival with favorable neurological status [7].

According to current Advanced Cardiac Life Support (ACLS) guidelines, the cornerstone pharmaceutical agents used during cardiac arrest include epinephrine, amiodarone and lidocaine [8]. Vasopressin was previously included in the ACLS algorithm as an alternative to epinephrine, but it was removed in the 2015 guideline update due to a lack of evidence of survival benefits [9]. During cardiac arrest, systemic perfusion is profoundly reduced, which significantly alters drug pharmacokinetics (PK) and may compromise the efficacy of intravenously administered medications [10,11]. To ensure timely drug delivery, current guidelines recommend establishing peripheral intravenous (IV) access as the first-line route. In cases where IV access is delayed or not feasible, intraosseous (IO) access is recommended as an effective alternative for rapid administration [8]. Historically, endotracheal (ET) drug administration was considered as a last-resort route when IV and IO access were unavailable, but it was phased out in favor of IO access due to unpredictable absorption and dosing inconsistencies [12]. Similarly, intracardiac injection of adrenaline was once explored but has since been abandoned due to practical limitations and risks. Establishing an IO access offers a rapid and effective route of drug administration, nevertheless concerns remain regarding its potentially slower or less efficient systemic absorption compared to IV delivery [13]. Experimental studies in porcine models of cardiac arrest have shown that maximum plasma concentration (C_max_) was significantly lower and the time to this peak concentration (T_max_) longer via tibial IO administration compared to IV routes—despite similar rates of ROSC [14,15]. Furthermore, clinical evidence remains inconsistent. A meta-analysis by Saad et al. found no significant differences between the two drug administration routes regarding 30-day survival, ROSC or favorable neurological outcome [14]. In contrast, a meta-analysis by Tabowei et al. reported that IV access was associated with significantly better outcomes regarding ROSC, survival to discharge and favorable neurological symptoms compared to IO access [15].

Common IO access sites include the proximal tibia (tibial IO-TIO), the proximal humeral head (humeral IO-HIO), and the sternum (sternal IO-SIO) [16,17,18,19]. However, current evidence regarding the optimal IO access site during resuscitation remains heterogeneous, with studies reporting variable outcomes. Notably, Burgert et al. suggest that proximal IO access sites, such as the SIO and HIO, should be considered in order to rapidly achieve effective drug concentrations during resuscitation [20]. Although PK equivalence between the IO and IV routes for epinephrine administration has not been demonstrated, it was indicated that the SIO route achieves serum epinephrine concentrations more closely approximating those of IV administration than the TIO route [20]. In support of the clinical utility of IO access, Beaumont et al. reported that both the TIO and HIO routes provide rapid and reliable access for the administration of epinephrine in a swine cardiac arrest model [21]. Furthermore, another randomized swine study highlighted the importance of adequate circulating volume for effective IO drug delivery [18]. Specifically, PK outcomes following HIO administration were inferior to those of IV administration under hypovolemic conditions, whereas no significant difference was observed under normovolemic conditions [18].

Although several large scale clinical trials and meta-analyses have investigated IO and IV access in relation to ROSC, 30-day survival, survival to discharge, and favorable neurological outcomes, the existing evidence remains largely focused on clinical endpoints [14,15,22,23]. To date, no comprehensive study or systematic review has evaluated the comparative PK (C_max_, T_max_) and pharmacodynamic (PD) (frequency of ROSC, time to ROSC, odds of ROSC) effects of various IO access routes (TIO, HIO, SIO)—both in comparison with one another and in direct comparison with the IV route and the ET route. These factors may ultimately influence guideline recommendations and guide evidence-based decisions during resuscitation.

Therefore, this systematic review and meta-analysis aims to synthesize available PK (e.g., C_max_, T_max_) and PD (e.g., frequency of ROSC, time to ROSC, odds of ROSC) data on the administration of specific drugs during cardiopulmonary resuscitation (CPR), with a focus on the impact of injection site. The study will include data from both human subjects and animal models, focusing on vasoactive and antiarrhythmic agents such as adrenaline, amiodarone, vasopressin and lidocaine. Comparisons will be made between different routes of drug administration (e.g., peripheral IV, central IV, SIO, HIO, TIO, ΕΤ) and under varying haemodynamic conditions (e.g., traumatic, hypovolemic, normovolemic CPR scenarios). Primary and secondary outcomes will include alterations in PK parameters (C_max_, T_max_) as well as PD outcomes (frequency of ROSC, time to ROSC and odds of ROSC). Thus, this review aims to clarify whether the choice of injection access site during CPR significantly influences drug effectiveness and clinical outcomes.

## 2. Materials and Methods

This study was conducted as a systematic review and meta-analysis in accordance with the Preferred Reporting Items for Systematic Review and Meta-Analyses (PRISMA) statement [24] (Supplementary Table S1). The study protocol was prospectively registered in the PROSPERO database (Registration Number: CRD420251112029).

### 2.1. Data Sources and Searches

Two independent reviewers (SCZ and SG) conducted a comprehensive literature search of PubMed, CENTRAL (Cochrane Central Register of Controlled Trials) and ClinicalTrias.gov, from database inception to August, 2025. Any discrepancies or disagreements were resolved by consensus with a third reviewer (EK).

The search aimed to identify studies comparing different injection sites for the same drug (antiarrhythmic and vasoactive, such as adrenaline/epinephrine, amiodarone, lidocaine and vasopressin) during CPR. Eligible studies included those using central or peripheral IV routes, ΕΤ and IO access in human, animal, or simulation models under various haemodynamic conditions (e.g., normovolemia, hypovolemia—traumatic).

Additionally, references of the included studies were manually screened for further eligible publications. Only studies published in English were included. Duplicate records were removed using Endnote 20 (Clarivate Analytics, Philadelphia, PA, USA). The complete search strategy is presented in Supplementary Table S2.

### 2.2. Eligibility Criteria

We included experimental and observational studies reporting PK and/or PD data relevant to the administration of resuscitation drugs during CPR.

Inclusion criteria: [a] Studies involving patients having undergone CPR or animal models (e.g., swine, dogs) simulating CPR—Swine models were considered eligible due to their physiological similarity to humas, particularly in cardiovascular dynamics, which makes them widely accepted model in preclinical CPR research. This focus on such models in preclinical studies may limit generalizability, and is further addressed in the Limitations section. [b] Both in-hospital and out-of-hospital CPR settings (or laboratory-based CPR for animals). [c] Only studies that compare different injection sites. [d] No date restrictions were applied. [e] Studies including adult subjects or animals. [f] Exogenous administration of resuscitation drugs (e.g., adrenaline/epinephrine, amiodarone, lidocaine, vasopressin). [g] Studies using artificial intelligence and simulation scenarios to model drug PK or PD during CPR.

Exclusion criteria: [a] Theoretical papers. [b] Case reports or single-patient case studies. [c] Studies that do not report PK or PD parameters. [d] Studies reporting only haemodynamic data without PK/PD analysis. [e] Studies involving paediatric populations. [f] Studies that do not include exogenous drug administration (e.g., studies measuring only endogenous catecholamines as stress markers without drug administration).

In cases of duplicate publications, the study with the largest sample size or longest follow-up was included.

### 2.3. Data Extraction

Data were extracted independently by two reviewers, SCZ and SG. When disagreements or conflicts occurred, a third reviewer (EK) was consulted to reach a consensus. Extracted data included study details (title, first author, year, location, design), clinical participant characteristics (type of subjects (humans or animals)), resuscitation drugs (e.g., adrenaline/epinephrine, amiodarone, lidocaine, vasopressin), haemodynamic status (normovolemic, hypovolemic conditions), PK (C_max,_ T_max_) and PD parameters (ROSC, time to ROSC, odds of ROSC). In the context of the included animal studies, “normovolemic” and “hypovolemic” cardiac arrest refer to experimental conditions in which cardiac arrest was included following controlled haemodynamic states. Normovolemia indicates the absence of prior volume depletion before arrest inductions, whereas hypovolemia is induced by fluid withdrawal prior to arrest. These terms describe the haemodynamic status preceding or present at the time of cardiac arrest in animal models. In clinical practice, hypovolemia is recognized as a reversible cause of cardiac arrest (one of the “4H’s and 4T’s”) and can contribute directly to the arrest event. In several instances, detailed PK datasets were not directly reported; instead, concentration–time profiles were presented in graphical form. These profiles were digitized using the GetData Graph Digitizer software (v.2.24), and subsequent non-compartmental analysis (NCA) was carried out employing the PK Solver add-in within Microsoft Excel (2016) to estimate the PK parameters.

### 2.4. Quality Assessment

Full texts were assessed for quality using the SYRCLE’s Risk of Bias tool for experimental studies, consisting of ten items specifically adapted to preclinical research, with each domain categorized as low, high, or unclear risk of bias. Human studies were assessed with Newcastle-Ottawa Scale (NOS) to evaluate the risk of bias, with a score of ≥7 indicating low bias. The Grading of Recommendations, Assessment, Development, and Evaluations (GRADE) approach was used to assess the degree of certainty of the evidence.

### 2.5. Outcome Measures

The primary objective was to assess PK and PD differences in resuscitation drug delivery based on injection site. Specifically, outcomes included C_max_, T_max_, frequency of ROSC, time to ROSC and odds of ROSC. Secondary analysis examined whether the haemodynamic status influenced drug effectiveness, and whether specific access routes were associated with optimal drug delivery. Hypovolemia and trauma-related states were evaluated in terms of hypovolemic shock, given that significant blood loss commonly underlies such conditions.

### 2.6. Statistical Analyses

To enhance the reliability of estimates for PK (C_max_, T_max_) and PD outcomes (frequency of ROSC, time to ROSC, odds of ROSC), a meta-analysis was carried out across eligible studies to derive a more precise overall effect size. A random-effects model was applied to pool effect estimates for the PK and PD parameters. Inclusion in the quantitative synthesis was restricted to studies reporting sufficient numerical data (e.g., sample size, mean values, and measures of variance). Investigations that did not provide the required data were excluded from statistical pooling but were still narratively summarized within the systematic review. Continuous variables were reported as mean ± standard deviation (SD). Summary estimates of continuous variables were reported as mean differences, and categorical variables as odds ratios (ORs) with 95% confidence intervals (CIs). The analyses were conducted in R using the “metafor” package to compute effect sizes, while between-study heterogeneity was quantified with the I^2^ statistic, with I^2^ > 50% indicating moderate-to-high heterogeneity. Results are presented as means accompanied by standard deviations (SDs). Results were visualized in a forest plot.

To account for physiological heterogeneity, we prespecified subgroup analyses by circulatory status and stratified studies as normovolemic or hypovolemic, with the latter including traumatic models in which animals underwent controlled blood withdrawal prior to the arrest protocol. Beyond reporting subgroup-specific pooled effects, we conducted meta-regression to formally test whether volume status modified the association between route and each outcome (C_max_, T_max_, time to ROSC, and ROSC). Models included fixed moderators for route (IV, humeral IO, tibial IO, sternal IO, endotracheal), volume status, and their interaction (route × status), were fit under random effects and results were expressed on the native scales (seconds for time outcomes, ng/mL for C_max_, and odds for ROSC). Statistical significance was set at *α* = 0.05.

In the meta-analysis, each study that reported more than one intraosseous (IO) route was further divided into separate comparisons between IV administration and each IO administration site. Practically, this means that a single multi-arm study contributed as many “sub-studies” as distinct IO routes (e.g., with sternal, tibial, humeral, and IV, the study yielded three contrasts: IV–sternal IO, IV–tibial IO, and IV–humeral IO). Consequently, the data for the IV administration were reused across several comparisons within the same study.

For the purposes of methodological consistency and accurate interpretation, all PK and PD variables were systematically converted into comparable measurement units.

## 3. Results

### 3.1. Literature Search

**Figure 1:**
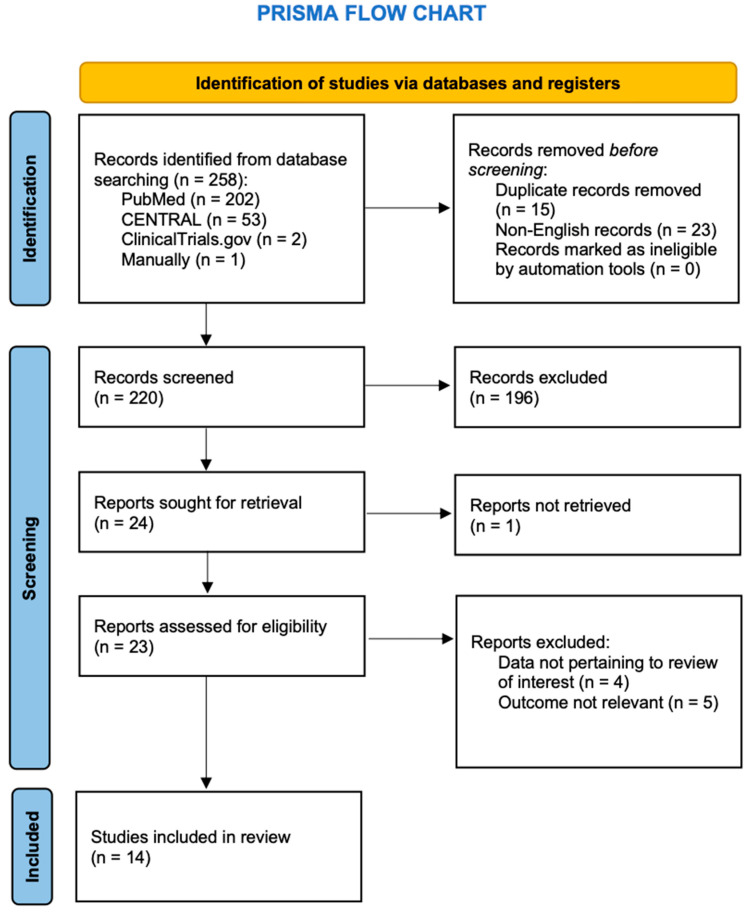
Flow diagram of the study (PRISMA flowchart).

A comprehensive literature search yielded a total of 258 records. After removing duplicates, 220 records remained for title and abstract screening. Among these, 196 records were excluded during the initial screening due to irrelevant outcomes or insufficient information. The full texts of 23 potentially eligible studies were assessed for inclusion. Following full-text review, 9 studies were excluded based on insufficient data and population mismatch. Finally, 14 studies met the inclusion criteria and were included in the systematic review. The study selection process is presented in the PRISMA Flowchart (Figure 1).

### 3.2. Study Characteristics and Quality Assessment

Among the initially identified records, 14 studies met the inclusion criteria and were included in this meta-analysis (Supplementary Table S3). All included studies were prospective experimental studies conducted on swine, published between 1992 and 2025, with sample sizes ranging from 15 to 49 animals per study [16,18,21,25,26,27,28,29,30,31,32,33,34,35]. No study involving human patients, simulations, or other animal species such as dogs was identified. Regarding the pharmacological interventions 9 studies investigated epinephrine, 3 investigated amiodarone, 2 investigated vasopressin and none investigated lidocaine. The routes of drug administration included peripheral intravenous (IV), IO—including HIO, SIO and TIO–as well as endotracheal (ET) routes. No study was identified that used central venous access. Finally, two haemodynamic statuses were reported across the included studies (normovolemia and hypovolemia—traumatic).

Quality assessments using the SYRCLE’s risk-of-bias tool revealed that most included studies adequately reported sequence generation, baseline characteristics, and allocation concealment, which were generally judged to be at a low risk of bias. In contrast, domains related to performance bias (random housing and blinding of caregivers/investigators) and detection bias (random outcome assessments and blinding of outcome assessors) were frequently scored as “unclear”, reflecting insufficient reporting rather than demonstrable methodological flaws. A small number of studies were judged to be at a high risk in specific domains, particularly caregiver blinding and blinding of outcome assessors. Attrition and reporting biases were consistently rated as low risk across studies, and no significant concerns were identified under “other sources of bias.” Overall, the assessment indicates that while selection procedures and reporting were generally robust, limited methodological detail in several studies—especially regarding blinding and housing—reduces the certainty of internal validity (Supplementary Table S4).

### 3.3. Meta-Analysis of PK During CPR in Normovolemia

**Figure 2:**
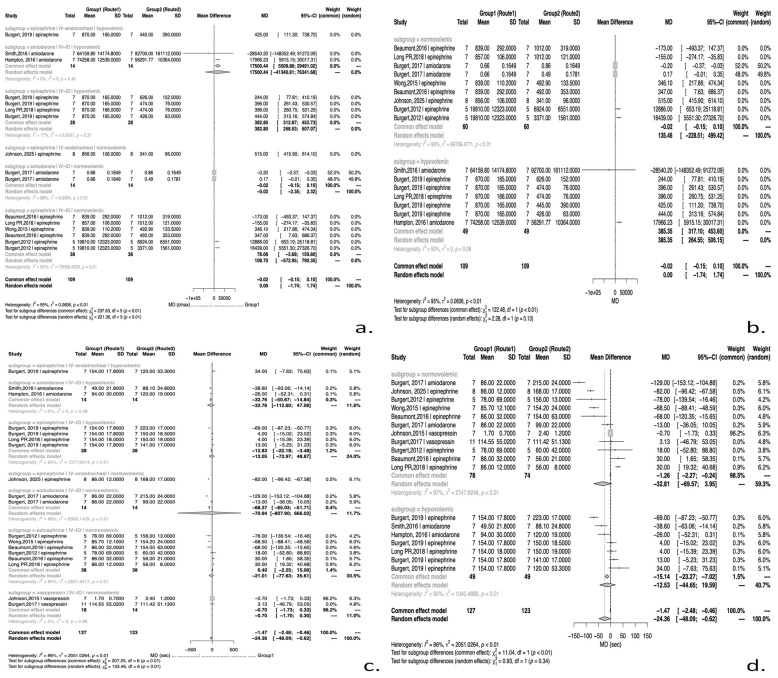
Forest plots of pharmacokinetic parameters during CPR with epinephrine, amiodarone and vasopressin under normovolemic and hypovolemic conditions. Panels (**a**,**b**) show pooled mean difference in C_max_ and panels (**c**,**d**) present pooled mean difference in T_max_. References appearing in the figure: [16,18,21,25,27,29,31,33,34].

This section presents the differences in C_max_ and T_max_ between three different routes (IO, IV, ET) of the same drug administration during CPR under normovolemic conditions (Figure 2).

#### 3.3.1. Meta-Analysis of C_max_ During CPR in Normovolemia

In the case of epinephrine, IV administration resulted in a lower peak concentration than HIO (IV vs. HIO pooled MD = −157.19 ng/mL; 95% CI −231.92 to −82.45; *p* = 0.0238), indicating significantly higher C_max_ with HIO. Conversely, TIO administration tended to yield lower C_max_ than IV, although the pooled estimates did not consistently reach statistical significance. While, IV administration of epinephrine is superior to ET administration, achieving higher peak concentrations (pooled MD = 515 ng/mL; 95% CI 415.90 to 614.10; *p* < 0.001).

For vasopressin, IV administration exceeded TIO (pooled MD = −32.09 ng/mL; 95% CI −54.92 to −9.25; *p* = 0.0059); however, these findings are supported by a single study.

Regarding amiodarone, IV administration appeared to result in higher peak concentration compared to TIO, without showing statistical significance (*p* > 0.05), while the comparison between tibial and sternal administration showed a significantly lower maximum concentration for the tibial route (pooled MD = −0.37 ng/mL; 95% CI −0.55 to −0.19; *p* < 0.001), indicating that sternal administration achieves higher peak levels. However, the data are likewise derived from a single study.

#### 3.3.2. Meta-Analysis of T_max_ During CPR in Normovolemia

Statistically significant differences were observed in T_max_ between different routes of administration for adrenaline and amiodarone during CPR under normovolemic conditions. Both epinephrine and amiodarone administrated via the TIO route were associated with a longer time to peak concentration compared to the IV route (pooled MD: 69.24 s; 95% CI 61.16 to 77.31; *p* < 0.001 and 129 s; 95% CI 104.88 to 153.12; *p* < 0.001, respectively). Likewise, T_max_ was significantly longer with tibial compared to sternal administration, for both epinephrine (pooled MD = 96 s; 95% CI 57.46 to 134.54; *p* < 0.001) and amiodarone (pooled MD = 116 s; 95% CI 91.88 to 140.12; *p* < 0.001). IV administration for epinephrine was, also, associated with a significantly shorter T_max_ compared with the humeral route (pooled MD = 30.00 s; 95% CI 29.99 to 30.00; *p* < 0.001). By contrast, no statistically significant difference in T_max_ was observed between IV and IO administration of vasopressin (pooled MD = −0.698 s; 95% CI −1.70 to 0.30; *p* = 0.0716).

### 3.4. Meta-Analysis of PK During CPR in Hypovolemia

This section presents a comparative analysis of C_max_ and T_max_ values resulting from different routes of drug administration during CPR under hypovolemic conditions (Figure 2).

#### 3.4.1. Meta-Analysis of C_max_ During CPR in Hypovolemia

Under hypovolemic conditions, IV administration resulted in significantly higher epinephrine exposure compared to IO routes overall (IV vs. IO pooled MD = +382.80 ng/mL; 95% CI: 258.53 to 507.07; *p* = 0.0022). Among different IO injection sites, the SIO route produced higher epinephrine concentrations than the TIO route (tibial vs. sternal MD = −200.00 ng/mL; 95% CI: −321.89 to −78.11; *p* = 0.0013). In addition, IV administration of epinephrine resulted in a significantly higher maximum concentration compared to ET administration; however, this finding is based on only one study (*p* = 0.0079).

#### 3.4.2. Meta-Analysis of T_max_ During CPR in Hypovolemia

Differences in T_max_ between administration routes were generally small and often not statistically significant in shock. A notable exception was observed for epinephrine between IV and IO administration, where IV exhibited a significantly longer T_max_ (pooled MD = 382.80 s; 95% CI: 258.53 to 507.07; *p* = 0.0022), indicating that IO administration reached peak concentration 6.4 min earlier.

Regarding amiodarone, the SIO route achieved a significantly shorter T_max_ compared to IV (MD, sternal − IV = −1.55 min; 95% CI −2.84 to −0.25; *p* = 0.0189), although data derived from a single study.

### 3.5. Comparison of PK in Normovolemia vs. Hypovolemia

This section compares the PK parameters (C_max_ and T_max_) of commonly used resuscitation drugs under normovolemic and hypovolemic conditions. The aim is to highlight potential differences in drug absorption and distribution due to circulating volume status.

#### 3.5.1. Comparison of C_max_ Between Normovolemia and Hypovolemia

In meta-regression, the overall main effect of shock status on epinephrine C_max_ was not statistically significant after adjustment (β = −180.71 ng/mL; *p* = 0.41). However, an exception was observed with TIO access for epinephrine regardless of haemodynamic status, which was associated with a significantly lower C_max_ compared to IV administration (β = −871.45 ng/mL; *p* = 0.0215).

#### 3.5.2. Comparison of T_max_ Between Normovolemia and Hypovolemia

The adjusted main effect of hypovolemia on epinephrine T_max_ was not statistically significant (β = −26.98 s; *p* = 0.36). However, after adjustment, TIO access for epinephrine was associated with a significantly longer T_max_ compared to IV administration (β = +106.14 s; *p* = 0.0042). In contrast, SIO access was significantly faster than TIO (β = +123.95 s; 95% CI: 54.13 to 193.77; *p* = 0.0035). Although HIO administration for epinephrine resulted in a shorter T_max_ compared to IV and ET administration was slower than IV, neither difference reached statistical significance (*p >* 0.05). In addition, meta-regression demonstrated significantly prolonged T_max_ for epinephrine under hypovolemic versus normovolemic conditions across several route comparisons: IV vs. humeral (β = +142.00 s; *p* < 0.001), tibial vs. IV (β = +116.24 s; *p* < 0.001), and tibial vs. sternal (β = +130.00 s; *p* < 0.001).

For amiodarone, meta-regression suggested an increased T_max_ under hypovolemia (β = +89.74 s; *p* = 0.0100).

### 3.6. Meta-Analysis of PD During CPR in Normovolemia

#### 3.6.1. Probability of ROSC

In normovolemic models receiving epinephrine, IO access was associated with significantly higher odds of achieving ROSC compared to IV (IV vs. IO pooled OR = 0.217; 95% CI: 0.07 to 0.62; *p* = 0.0134), with HIO contributing most consistently to this effect (*p <* 0.001).

#### 3.6.2. Time to ROSC

In epinephrine administration under normovolemic conditions, time to ROSC favored IO over IV (IV vs. IO pooled MD = +177.52 s; 95% CI 134.40 to 220.64; *p* = 0.0032), indicating a more rapid clinical response with IO delivery in normal volume status. In particular, ΤIO administration was associated with a markedly shorter time to ROSC compared with IV (pooled MD = –172.16 s; 95% CI −173.16 to −171.17; *p* < 0.001).

### 3.7. Meta-Analysis of PD During CPR in Hypovolemia

#### 3.7.1. Probability of ROSC

In hypovolemic models, no statistically significant differences in frequency of ROSC were observed between adrenaline and amiodarone administered via different routes during CPR. For epinephrine, intravenous and intraosseous administration yielded comparable outcomes (OR = 1.015; 95% CI 0.305 to 3.374; *p* = 0.9744). Likewise, no significant differences were detected among individual intraosseous access sites, including humeral (OR = 0.811; 95% CI 0.029 to 22.23; *p* = 0.811), tibial (OR = 1.00; 95% CI 0.12 to 8.31; *p* = 1.00), and sternal routes (OR = 1.78; 95% CI 0.21 to 14.77; *p* = 0.5942). Similarly, for amiodarone, IV and IO administration demonstrated comparable effectiveness under hypovolemic conditions (OR = 0.687; 95%CI 2.79 to 168.96; *p* = 0.5456), with no significant variation across tibial (OR = 2.40; 95% CI 0.16 to 34.93; *p* = 0.5217) or sternal access (OR = 1.00; 95% CI 0.098 to 10.17; *p* = 1.00).

#### 3.7.2. Time to ROSC

IV administration of epinephrine achieved a shorter time to ROSC compared to HIO access (pooled MD: −291 s; 95% CI: −532.77 to −49.23; *p* = 0.0183), with ROSC being achieved approximately 4.9 min earlier with IV administration of adrenaline. Moreover, the time to ROSC was shorter with SIO access than TIO (pooled MD: 122 s; 95% CI 28.12 to 215.88; *p* = 0.0109). Both results were derived from a single study.

For amiodarone, IV administration achieved a shorter time to ROSC compared to IO routes (pooled MD: −113.53 s, 95% CI: −176.75 to −50.31, *p* = 0.0279), corresponding to 1.9 min earlier.

### 3.8. Comparison of PD in Normovolemia vs. Hypovolemia

Shock status independently reduced the odds of ROSC when epinephrine was administered (β ≈ −1.64; SE: 0.57; *p* = 0.015). A significant interaction between administration route and haemodynamic state was observed for time to ROSC following epinephrine administration, when comparing IV with HIO access. HIO was associated with shorter time to ROSC under normovolemia, whereas IV administration of adrenaline in a state of hypovolemia resulted in shorter time to ROSC (interaction *p* = 0.0183 < 0.05), reflecting an approximate 5–6-min difference between states.

**Figure 3:**
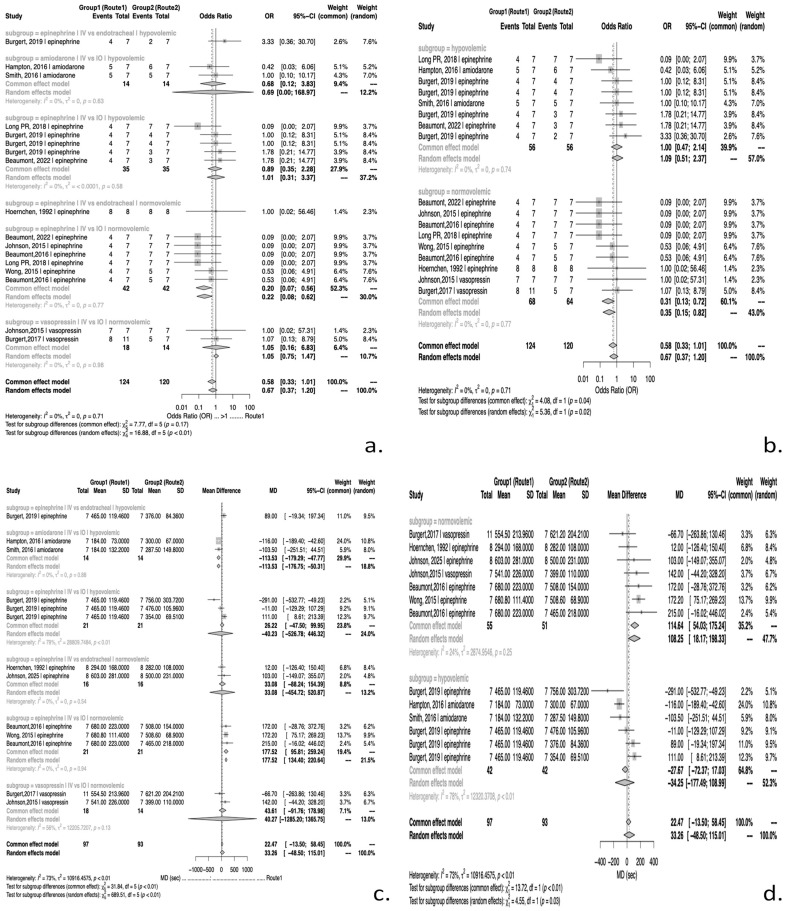
Forest plots of pharmacodynamic outcomes during CPR with epinephrine, amiodarone and vasopressin under normovolemic and hypovolemic conditions. Panels (**a**,**b**) present the probability of ROSC and panels (**c**,**d**) illustrate time to ROSC. References appearing in the figure: [16,18,21,26,27,28,30,31,33,34].

A comparative forest plot summarizing the pharmacodynamic effects of epinephrine, amiodarone and vasopressin on ROSC probability and time to ROSC under normovolemic and hypovolemic conditions is presented in Figure 3.

### 3.9. Summary of Drug Delivery Routes in Swine Cardiac Arrest Models

Across the included swine cardiac-arrest models, both PK and PD were substantially influenced by the route of administration.

**Epinephrine.** In normovolemic models, HIO access yielded greater and faster epinephrine exposure than IV administration (significantly higher C_max_; *p* = 0.0238 < 0.05 and shorter T_max_; *p* < 0.001) and translated into shorter time to ROSC (*p* = 0.0681). Conversely, TIO administration tended to be absorbed more slowly than IV (*p* < 0.001). ET access also underperformed compared to IV administration regarding both exposure and speed, with both differences reaching statistical significance.

In a state of hypovolemia, IV administration regained the advantage for epinephrine exposure against IO (MD = +382.80 ng/mL; *p* = 0.0022) and time to ROSC specifically against via HIO (IV vs. HIO: ~5–6 min faster) (*p* = 0.0183 < 0.05). Among IO sites, SIO achieved higher concentrations than TIO (*p* = 0.0013). Notably, TIO access under hypovolemia was associated with significantly lower C_max_ compared to IV (β = −871.45 ng/mL; *p* = 0.0215). SIO also performed favorably, with significantly shorter time to ROSC than TIO (*p* = 0.0109).

**Amiodarone.** Under normovolemia IV administration consistently resulted in higher or faster exposure compared to IO routes, although statistical significance was not always achieved. However, during shock, SIO access achieved a significantly shorter T_max_ than IV (MD = −1.55 s; *p* = 0.0189), based on a single study. Time to ROSC following amiodarone was also significantly shorter with IV than with IO access (pooled MD = −113.53 s; *p* = 0.0279), corresponding to a 1.9-min faster response, with TIO contributing most consistently to this effect (*p <* 0.05). Furthermore, meta-regression suggested that hypovolemia significantly increased T_max_ (β = +89.74 s; *p* = 0.0100).

**Vasopressin.** For vasopressin, IV administration achieved significantly higher C_max_ than TIO access under normovolemia (MD = −32.09 ng/mL; *p* = 0.0059), although this result was derived from a single study. No statistically significant differences in ROSC frequency were observed among the different administration routes in hypovolemic models.

## 4. Discussion

This systematic review and meta-analysis highlights the pivotal influence of injection site on the PK and PD of resuscitation drugs administered during cardiac arrest in swine models. The results demonstrate that both the volume status (normovolemia vs. hypovolemia) and injection site (IV, IO-SIO, HIO, TIO-, and ET) affect drug absorption, systemic exposure, and ultimately, clinical outcomes such as ROSC.

In normovolemic animal models, HIO access has demonstrated greater and faster systemic exposure to epinephrine compared to IV administration, which translated into shorter time to ROSC and higher ROSC probability. However, this evidence is not consistently supported by the broader literature, where several studies report no significant difference between IO and IV routes or better results via IV administration [3,15,36]. This discrepancy may be attributed to the limited number of studies and small sample sizes currently available, thus rendering the results unsuitable for extrapolation.

In our study, ET administration of epinephrine underperformed compared to the IV route in terms of both systematic exposure and speed of onset. Although ET access was historically considered an alternative when IV route was unavailable, its disadvantages in PK and inconsistent efficacy no longer support its use in current clinical practice [37]. Reflecting these concerns, ET administration was removed from the American Heart Association (AHA) ACLS guidelines in 2015, whereas, in the same guidelines, IV or IO access became the recommended routes for drug delivery during cardiac arrest [9].

In normovolemic conditions, SIO access demonstrated superior outcomes relative to TIO access. These findings align with the review of the existing literature indicating that the proximal IO injection sites—such as HIO and SIO—achieve higher and faster serum concentrations, more similar to IV administration, compared to distal injection sites [20]. Available anatomical and physiological data support this mechanism, noting that the HIO and SIO sites are connected to the central venous system via larger, more direct venous pathways such as the subclavian and internal thoracic veins [38]. These pharmacokinetic advantages and anatomical characteristics underscore the clinical relevance of selecting proximal IO access sites during resuscitation.

Under hypovolemic conditions, IV administration achieved a statistically significantly higher C_max_ compared to IO routes, reaffirming its pharmacokinetic superiority. Conversely, IO administration was associated with a significantly faster T_max_, yet a markedly lower C_max_ relative to IV. These findings likely reflect the pathophysiological effects of hypovolemic shock, where peripheral perfusion is markedly reduced [39]. IO drug delivery depends on medullary venous drainage, which may become severely compromised under low-flow states, thereby impairing systemic drug absorption [40,41]. In contrast, peripheral IV access may provide more reliable delivery in this setting, probably because it is not affected by the impaired medullary circulation observed during hypovolemia. Although the IO route remains a valuable alternative in various shock states—especially as an immediate option when IV access is not yet available—it is often used in order to gain time until a more definitive IV route can be established [40]. This finding warrants further investigation, as it may have important implications for the clinical approach to drug administration during cardiac arrest in hypovolemic shock.

Additionally, recent clinical data suggest that adrenaline dose itself can significantly influence resuscitation outcomes, regardless of the administration route. A retrospective study of 508 OHCA patients found that lower adrenaline doses were associated with better ROSC rates and that a multivariable model including demographics and pandemic status predicted ROSC more accurately than adrenaline dose alone (AUC 0.773 vs. 0.711) [42].

### Limitations and Future Directions

This systematic review and meta-analysis is limited by the exclusive reliance on swine models, which—while physiologically comparable to humans—may not fully capture the complexity of human PK and PD responses during cardiac arrest. Moreover, the classification of cardiac arrest as normovolemic or hypovolemic reflects controlled experimental condition in animal models and may not fully represent the complex clinical scenarios in which arrest occurs. Additionally, although the ET route and vasopressin are no longer recommended in current ACLS protocols, they were included due to the availability of existing data in the literature. Another limitation is the relatively small number of eligible studies, which may affect the generalizability and statistical power of the findings. Furthermore, the inclusion of both vasopressors and antiarrhythmics, rather than focusing on one or two specific drugs, reflects the limited data available but introduces heterogeneity that should be considered when interpreting results. This highlights a need for further high-quality studies in this area.

Data on PK and PD following drug administration via central venous catheters (CVC) in swine cardiac arrest models are lacking in the current literature. This represents an important gap, as CVCs are commonly used in clinical practice and may provide different PK/PD profiles compared to peripheral or IO routes. Future preclinical studies should investigate the PK and PD characteristics of resuscitation drugs administered through CVC to better inform clinical protocols.

Nevertheless, the investigation of PK and PD across different injection sites during cardiac arrest in human subjects remains inherently challenging due to ethical and practical constraints. Furthermore, to date there is a lack of literature examining these dynamics in simulation scenarios, which could offer valuable insight while circumventing these limitations.

## 5. Conclusions

In conclusion, the injection site significantly influences the PK and PD of epinephrine during cardiac arrest. Proximal IO routes may offer advantages under normovolemic conditions due to faster and more effective systemic drug absorption, while IV access appears superior in hypovolemic shock, likely due to impaired medullary perfusion affecting IO efficacy. Although the IO route remains a valuable immediate option—particularly when IV access is delayed—its effectiveness depends on both the anatomical site and the volume status.

Further research is needed to refine drug delivery strategies in varying hemodynamic conditions and to explore under-investigated routes such as central venous access, especially in hypovolemic states.

## Data Availability

The data underlying this article will be shared on reasonable request by the corresponding author.

## Supplementary Materials

The following supporting information can be downloaded at https://www.mdpi.com/article/10.3390/jcm14217497/s1. Table S1: Preferred Reporting Items for Systematic reviews and Meta-Analysis (PRISMA) checklist; Table S2: Search Terms; Table S3: Study Characteristics; Table S4: Quality Assessment; Table S5a: Pharmacokinetic Study Characteristics; Table S5b: Pharmacodynamic Study Characteristics; Table S6: Meta-analysis for Continuous Outcomes; Table S7: Meta-analysis for Dichotomous Outcomes; Table S8: Meta-regression Table for Pharmacokinetic Outcomes; Table S9: Meta-regression Table for Pharmacodynamic Outcomes; Table S10: Meta-analysis for continuous outcomes (Subgroup Analysis); Table S11: Meta-analysis for Dichotomous Outcomes (Subgroup Analysis); Table S12: Metaregression Table for Pharmacokinetic Outcomes (Subgroup Analysis); Table S13: Meta-regression Table for Pharmacodynamic Outcomes (Subgroup Analysis).

## Abbreviations

The following abbreviations are used in this manuscript:

IV	intravenous
IO	intraosseous
ET	endotracheal
PK	pharmacokinetic
PD	pharmacodynamic
CPR	cardiopulmonary resuscitation
ORs	odds ratio
Cis	confidence intervals
HIO	humeral intraosseous
C_max_	maximum concentration
T_max_	time to maximum concentration
ROSC	return of spontaneous circulation
SIO	sternal intraosseous
TIO	tibial intraosseous
OHCA	out-of-hospital cardiac arrest
IHCA	in-hospital cardiac arrest
ACLS	Advanced Cardiac Life Support
CVC	central venous catheters
AHA	American Heart Association
