# Supplementary material for: Does the Injection Site Matter During CPR? A Systematic Review and Meta-Analysis of Drug Pharmacokinetics and Pharmacodynamics
Source: J Clin Med. 2025 Oct 23;14(21):7497. doi: 10.3390/jcm14217497 (PMC12609464; doi:10.3390/jcm14217497)
Supplement: Supplementary file 1 [file jcm-14-07497-s001.zip › jcm-3927608-supplementary.pdf]

Supplementary Material:

Does Injection Site Matter During CPR? A Systematic Review and Meta-analysis of Drug Pharmacokinetics and Pharmacodynamics.

Contents

Supplementary Table 1: Preferred Reporting Items for Systematic reviews and Meta-Analysis (PRISMA) checklist 2

Supplementary Table 2: Search Terms 5

Supplementary Table 3: Study Characteristics 6

Supplementary Table 4: Quality Assessment 8

Supplementary Table 5a: Pharmacokinetic Study Characteristics 11

Supplementary Table 5b: Pharmacodynamic Study Characteristics 13

Supplementary Table 6: Meta-analysis for Continuous Outcomes 15

Supplementary Table 7: Meta-analysis for Dichotomous Outcomes 17

Supplementary Table 8: Meta-regression Table for Pharmacokinetic Outcomes 18

Supplementary Table 9: Meta-regression Table for Pharmacodynamic Outcomes 19

Supplementary Table 10: Meta-analysis for continuous outcomes (Subgroup Analysis) 20

Supplementary Table 11: Meta-analysis for Dichotomous Outcomes (Subgroup Analysis) 23

Supplementary Table 12: Metaregression Table for Pharmacokinetic Outcomes (Subgroup Analysis) 24

Supplementary Table 13: Meta-regression Table for Pharmacodynamic Outcomes (Subgroup Analysis) 26

Supplementary Table 1: Preferred Reporting Items for Systematic reviews and Meta-Analysis (PRISMA) checklist

Section and Topic	Item #	Checklist item	Location where item is reported
**TITLE**			
Title	1	Identify the report as a systematic review.	1
**ABSTRACT**			
Abstract	2	See the PRISMA 2020 for Abstracts checklist.	1-2
**INTRODUCTION**			
Rationale	3	Describe the rationale for the review in the context of existing knowledge.	2-3
Objectives	4	Provide an explicit statement of the objective(s) or question(s) the review addresses.	3
**METHODS**			
Eligibility criteria	5	Specify the inclusion and exclusion criteria for the review and how studies were grouped for the syntheses.	4
Information sources	6	Specify all databases, registers, websites, organisations, reference lists and other sources searched or consulted to identify studies. Specify the date when each source was last searched or consulted.	3-4
Search strategy	7	Present the full search strategies for all databases, registers and websites, including any filters and limits used.	3-4
Selection process	8	Specify the methods used to decide whether a study met the inclusion criteria of the review, including how many reviewers screened each record and each report retrieved, whether they worked independently, and if applicable, details of automation tools used in the process.	4
Data collection process	9	Specify the methods used to collect data from reports, including how many reviewers collected data from each report, whether they worked independently, any processes for obtaining or confirming data from study investigators, and if applicable, details of automation tools used in the process.	4
Data items	10a	List and define all outcomes for which data were sought. Specify whether all results that were compatible with each outcome domain in each study were sought (e.g. for all measures, time points, analyses), and if not, the methods used to decide which results to collect.	5
	10b	List and define all other variables for which data were sought (e.g. participant and intervention characteristics, funding sources). Describe any assumptions made about any missing or unclear information.	5
Study risk of bias assessment	11	Specify the methods used to assess risk of bias in the included studies, including details of the tool(s) used, how many reviewers assessed each study and whether they worked independently, and if applicable, details of automation tools used in the process.	4
Effect measures	12	Specify for each outcome the effect measure(s) (e.g. risk ratio, mean difference) used in the synthesis or presentation of results.	5
Synthesis methods	13a	Describe the processes used to decide which studies were eligible for each synthesis (e.g. tabulating the study intervention characteristics and comparing against the planned groups for each synthesis (item #5)).	5
	13b	Describe any methods required to prepare the data for presentation or synthesis, such as handling of missing summary statistics, or data conversions.	5

Section and Topic	Item #	Checklist item	Location where item is reported
	13c	Describe any methods used to tabulate or visually display results of individual studies and syntheses.	5
	13d	Describe any methods used to synthesize results and provide a rationale for the choice(s). If meta-analysis was performed, describe the model(s), method(s) to identify the presence and extent of statistical heterogeneity, and software package(s) used.	5
	13e	Describe any methods used to explore possible causes of heterogeneity among study results (e.g. subgroup analysis, meta-regression).	5
	13f	Describe any sensitivity analyses conducted to assess robustness of the synthesized results.	5
Reporting bias assessment	14	Describe any methods used to assess risk of bias due to missing results in a synthesis (arising from reporting biases).	4
Certainty assessment	15	Describe any methods used to assess certainty (or confidence) in the body of evidence for an outcome.	4
**RESULTS**			
Study selection	16a	Describe the results of the search and selection process, from the number of records identified in the search to the number of studies included in the review, ideally using a flow diagram.	6
	16b	Cite studies that might appear to meet the inclusion criteria, but which were excluded, and explain why they were excluded.	6
Study characteristics	17	Cite each included study and present its characteristics.	7
Risk of bias in studies	18	Present assessments of risk of bias for each included study.	7
Results of individual studies	19	For all outcomes, present, for each study: (a) summary statistics for each group (where appropriate) and (b) an effect estimate and its precision (e.g. confidence/credible interval), ideally using structured tables or plots.	7-14
Results of syntheses	20a	For each synthesis, briefly summarise the characteristics and risk of bias among contributing studies.	7
	20b	Present results of all statistical syntheses conducted. If meta-analysis was done, present for each the summary estimate and its precision (e.g. confidence/credible interval) and measures of statistical heterogeneity. If comparing groups, describe the direction of the effect.	7-14
	20c	Present results of all investigations of possible causes of heterogeneity among study results.	7
	20d	Present results of all sensitivity analyses conducted to assess the robustness of the synthesized results.	10-11
Reporting biases	21	Present assessments of risk of bias due to missing results (arising from reporting biases) for each synthesis assessed.	7
Certainty of evidence	22	Present assessments of certainty (or confidence) in the body of evidence for each outcome assessed.	7-14
**DISCUSSION**			
Discussion	23a	Provide a general interpretation of the results in the context of other evidence.	14-15
	23b	Discuss any limitations of the evidence included in the review.	15
	23c	Discuss any limitations of the review processes used.	15

Section and Topic	Item #	Checklist item	Location where item is reported
	23d	Discuss implications of the results for practice, policy, and future research.	15
**OTHER INFORMATION**			
Registration and protocol	24a	Provide registration information for the review, including register name and registration number, or state that the review was not registered.	15-16
	24b	Indicate where the review protocol can be accessed, or state that a protocol was not prepared.	15-16
	24c	Describe and explain any amendments to information provided at registration or in the protocol.	15-16
Support	25	Describe sources of financial or non-financial support for the review, and the role of the funders or sponsors in the review.	15-16
Competing interests	26	Declare any competing interests of review authors.	15-16
Availability of data, code and other materials	27	Report which of the following are publicly available and where they can be found: template data collection forms; data extracted from included studies; data used for all analyses; analytic code; any other materials used in the review.	15-16

From: Page MJ, McKenzie JE, Bossuyt PM, Boutron I, Hoffmann TC, Mulrow CD, et al. The PRISMA 2020 statement: an updated guideline for reporting systematic reviews. BMJ 2021;372:n71. doi: 10.1136/bmj.n71. This work is licensed under CC BY 4.0. To view a copy of this license, visit https://creativecommons.org/licenses/by/4.0/

Supplementary Table 2: Search Terms

**Date 10.07.2025**		
Search entry Pubmed, MEDLINE		
Search	Entry terms	Result
#1	"pharmacokinetics" OR "drug metabolism" OR "distribution" OR "elimination"	1,880,468
#2	"cardiopulmonary resuscitation" OR "cardiac arrest" OR "CPR"	76,910
#3	"epinephrine" OR "amiodarone" OR "lidocaine" OR "anesthetics" OR "sedatives"	239,574
#4	#1 AND #2 AND #3	202

Supplementary Table 3: Study Characteristics

No.	Authors	Year of publication	Country	Sample size	Haemodynamical status	Administered Drugs	Dosage	Timing of Administration	Route/Site of Administration	Pharmacokinetic and Pharmacodynamic outcomes	Conclusions *statistically significant
1	Johnson D., et al. [27]	2025	USA	40 (n=8 per group)	Normovolemia	Epinephrine	0.1mg/kg ET; 1mg IV; 2mg ET	Repeated every 4 min	ET; IV	C_{max}, T_{max}, AUC, plasma mean concentration over 4 min, odds of ROSC, time to ROSC	C_{max} and AUC higher in the IV group compared to the 0,1mg ET group. T_{max} longer in the 0,1mg ET that the IV group. ROSC frequency higher in 0,1mg ET group. There was highlighted that weight-based dosing of 0.1mg/kg of epinephrine by the ET should be used as a first-line intervention.
2	Burgert J., et al. [16]	2019	USA	49 (n=7 per group)	Hypovolemia	Epinephrine	1mg	Repeated every 4 min	ET; TIO; SIO; HIO; IV	C_{max}, T_{max}, plasma mean concentration over 5 min, frequency and time to ROSC, odds of ROSC	C_{max} of IV group higher than the TIO group. Significant differences in ROSC existed between all groups and the CPR only group. In this traumatic cardiac arrest experimental model, the ET absorption was variable and unreliable compared to IV and IO route.
3	Beaumont D., et al. [26]	2022	USA	28 (n=7 per group)	Normovolemia and Hypovolemia	Epinephrine	1mg	Repeated every 4 min	HIO and IV in two groups (normovolemic and hypovolemic)	AUC, frequency and odds of ROSC	The AUC in the HIO hypovolemic group was significantly less than both HIO normovolemic group and IV hypovolemic. HIO normovolemic group higher occurrence of ROSC compared to HIO and IV hypovolemic group. HIO an effective administration route for normovolemic, but not in hypovolemic group.
4	Wong M., et al. [27]	2015	USA	21 (n=7 per group)	Normovolemia	Epinephrine	1mg	Repeated every 4 min	TIO; IV	C_{max}, T_{max}, ROSC and time to ROSC	Significant difference between IV group and TIO group in epinephrine concentrations at specific time intervals: 60, 90 and 120 sec. Concluded that TIO access should be considered relevant to IV access.
5	Johnson D., et al. [28]	2015	USA	21 (n=7 per group)	Normovolemia	Epinephrine	1mg	Repeated every 4 min	HIO; IV	C_{max}, T_{max}, (data only in charts) ROSC and odds of survival	HIO delivers higher concentration than the IV route at 30 sec. HIO may be considered as an alternative route to CA.
6	Burgert J., et al. [29]	2012	USA	15 (n=5 per group)	Normovolemia	Epinephrine	1mg	Repeated every 4 min	TIO; SIO; IV	C_{max}, T_{max}	C_{max} of epinephrine is higher when administered through IV compared with both TIO and SIO. Time to peak concentration was similar in IV and SIO, but delayed in TIO group.

**7**	Beaumont D., et al. [21]	2016	USA	28 (n=7 per group)	Normovo-lemia	Epinephrine	1mg	Repeated every 4 min	TIO; HIO; IV	C_{max}, T_{max}, ROSC, time to ROSC, mean concentration over time and odds ratio	C_{max} in HIO group was significantly higher than the TIO group. The T_{max} was significantly shorter for both the IV and HIO versus the TIO group. The TIO and HIO groups proposed as reliable routes during cardiac arrest.
**8**	Long LRP., et al. [18]	2018	USA	28 (n=7 per group)	Normovo-lemia and Hypovole-mia	Epinephrine	1mg	Repeated every 4 min	HIO normovolemia; HIO hypovolemia; IV normovolemia; IV hypovolemia	C_{max}, T_{max}, ROSC, time to ROSC (data only in chart), mean concentration over time and odds ratio	C_{max} was significantly higher, the T_{max} and the time to ROSC were significantly faster in the HIO normovolemic compared to the HIO hypovolemic group. The HIO is an effective route in a normovolemic model.
**9**	Hornchen U., et al. [30]	1992	Germany	24 (n=8 per group)	Normovo-lemia	Epinephrine	10µg/kg IV, 100 µg/kg EB diluted in 10ml of normal saline	n.m.	IV, EB	C_{max}, T_{max}, mean concentration over time, AUC, elimination half-life, absorption half-life, total clearance (not included in the meta-analysis due to the use of mixed venous and arterial blood samples), ROSC, time to ROSC	Additional IV or EB adrenaline medication resulted in higher arterial plasma concentrations, improved circulation during CPR, and better resuscitability.
**10**	Hampton K., et al. [31]	2016	USA	28 (n=7 per group)	Normovo-lemia	Amiodarone	300mg	According to ACLS protocol	TIO, IV	C_{max}, T_{max}, ROSC, time to ROSC, mean concentration over time	The TIO a reliable alternative route during cardiac arrest
**11**	Burgert J., et al. [32]	2017	USA	21 (n=7 per group)	Normovo-lemia	Amiodarone	300mg	According to ACLS protocol	TIO, SIO, IV	C_{max}, T_{max}	TIO group took nearly three times longer to reach T_{max} than the SIO and IV groups. The SIO route was more effective than the TIO route.
**12**	Smith SS., et al. [33]	2016	USA	28 (n=7 per group)	Normovo-lemia	Amiodarone	300mg	According to ACLS protocol	SIO, IV	C_{max}, T_{max}, ROSC, time to ROSC, mean concentration over time	The SIO a reliable alternative route during cardiac arrest
**13**	Burgert JM., et al. [34]	2017	USA	27 (n=9 per group)	Normovo-lemia	Vasopressin	40U	After the second cycle of CPR	HIO, IV	C_{max}, T_{max}, occurrence, odds and time to ROSC	The HIO an effectively route during cardiac arrest, resuscitative and pharmacokinetics effects comparable to IV.

**14**	Johnson D., et al. [35]	2015	USA	21 (n=7 per group)	Normovo-lemia	Vasopressin	40U	After the second cycle of CPR	TIO, IV	C_{max}, T_{max}, occurrence, odds and time to ROSC	The C_{max} of IV group was higher compared to the TIO group. All subjects had ROSC in both IV and TIO groups. The TIO is an effective route in cardiac arrest model.

ET, endotracheal; IV, intravenous; Cmax, concentration maximum; T_{max}, time to C_{max}; AUC, area under the curve; ROSC, return of spontaneous circulation; TIO, tibial intraosseous; SIO, sternal intraosseous; HIO, humeral intraosseous; n.m., not mentioned; EB, endobronchial

Supplementary Table 4: Quality Assessment

Study	Selection bias: Sequence genera-tion	Selection bias: Baseline characteri-stics	Selection bias: Allocation conceal-ment	Performance bias: Random housing	Performance bias: Blinding of caregivers/inve-stigators	Detection bias: Random outcome assess-ment	Detection bias: Blinding of outcome assessor	Attrition bias: Incom-plete outcome data	Reporting bias: Selective outcome reporting	Other bias
Johnson D., et al. 2025 [25]	Low	Low	Low	Unclear	High	Unclear	Low	Low	Low	Low
Burgert J., et al. 2019 [16]	Low	Low	Low	Unclear	Unclear	Unclear	Unclear	Low	Low	Low
Beau-mont D., et al. 2022 [26]	Low	Low	Low	Unclear	Unclear	Unclear	High	Low	Low	Low
Wong M., et al. 2015 [27]	Low	Low	Low	Unclear	Unclear	Unclear	Unclear	Low	Low	Low
Johnson D., et al. 2015 [28]	Low	Low	Low	Unclear	Unclear	Unclear	Unclear	Low	Low	Low
Burgert J., et al. 2012 [29]	Low	Low	Low	Unclear	Unclear	Unclear	Unclear	Low	Low	Low
Beau-mont D., et al. 2016 [21]	Unclear	Low	Unclear	Unclear	Unclear	Unclear	High	Low	Low	Low

Long LRP., et al. 2018 [18]	Low	Low	Low	Unclear	Unclear	Unclear	Unclear	Low	Low	Low
Horn-chen U., et al. 1992 [30]	Unclear	Low	Unclear	Unclear	Unclear	Unclear	Unclear	Low	Low	Low
Hampton K., et al. 2016 [31]	Low	Unclear	Low	Unclear	Unclear	Unclear	High	Low	Low	Low
Burgert J., et al. 2017 [32]	Low	Low	Low	Unclear	Unclear	Unclear	Unclear	Low	Low	Low
Smith SS., et al. 2016 [33]	Low	Low	Low	Unclear	High	Unclear	Low	Low	Low	Low
Burgert JM., et al. 2017 [34]	Low	High	Low	Unclear	Unclear	Unclear	Unclear	Low	Low	Low
Johnson D., et al. 2015 [35]	Unclear	Low	Unclear	Unclear	Unclear	Unclear	Unclear	Low	Low	Low

Supplementary Table 5a: Pharmacokinetic Study Characteristics

Author, Year	Drug	Route/ Site of Administration	Number of Swine (n)	C_{max} mean ± sd (ng/mL)	T_{max} mean ± sd (sec)	Note
Johnson, 2025	Epinephrine	IV	8	856 ± 106	86 ± 12	Normovolemic
		Endotracheal	8	341 ± 96	168 ± 17	
Wong, 2015	Epinephrine	Tibial	7	492.9 ± 133.5	154.2 ± 24	Normovolemic
		IV	7	839 ± 110.2	85.7 ± 12.1	
Burgert, 2012	Epinephrine	Tibial	5	3371 ± 1561	156 ± 13	Normovolemic
		IV	5	19810 ± 12323	78 ± 69	
		Sternal	5	6924 ± 6551	60 ± 42	
Beaumont, 2016	Epinephrine	Tibial	7	492 ± 353	154 ± 63	Normovolemic
		IV	7	839 ± 292	86 ± 32	
		Humeral	7	1012 ± 319	56 ± 21	
Long PR, 2018	Epinephrine	IV	7	857 ± 106	86 ± 12	Normovolemic
		Humeral	7	1012 ± 121	56 ± 8	
Burgert, 2017	Amiodarone	Tibial	7	0.49118 ± 0.17807	215 ± 24	Normovolemic
		Sternal	7	0.8587 ± 0.16486	99 ± 22	
		IV	7	0.65854 ± 0.16487	86 ± 22	
Burgert, 2017	Vasopressin	IV	11	61.8537 ± 22.74504	114.55 ± 55.02	Normovolemic
		Humeral	7	71.7539 ± 26.74458	111.42 ± 51.13	
Johnson, 2015	Vasopressin	Tibial	7	38.630 ± 12.641	2.4 ± 1.2	Normovolemic
		IV	7	70.717 ± 28.118	1.7 ± 0.7	
Burgert, 2019	Epinephrine	Tibial	7	426 ± 63	223 ± 17	Hypovolemic
		IV	7	870 ± 165	154 ± 17.8	
		Sternal	7	626 ± 152	141 ± 17	
		Humeral	7	474 ± 76	150 ± 18.5	
		Endotracheal	7	445 ± 390	120 ± 53.3	
Long PR, 2018	Epinephrine	IV	7	870 ± 166	154 ± 18	Hypovolemic
		Humeral	7	474 ± 76	150 ± 19	
Hampton, 2016	Amiodarone	Tibial	7	56291.77 ± 10364	120 ± 19	Hypovolemic

		IV	7	74258 ± 12539	94 ± 30	
Smith, 2016	Amiodarone	Sternal	7	92700 ± 161112	88.1 ± 24.8	Hypovolemic
		IV	7	64159.8 ± 14174.8	49.5 ± 21.8	

Supplementary Table 5b: Pharmacodynamic Study Characteristics

Author, Year	Drug	Route/ Site of Administration	Number of Swine (n)	Time to ROSC mean ± sd (sec)	ROSC_success (n)	Rosc_failure (n)	Note
Johnson, 2025	Epinephrine	IV	8	603 ± 281	NA	NA	Normovolemic
		Endotracheal	8	500 ± 231	NA	NA	
Beaumont, 2022	Epinephrine	IV	7	NA	4	3	Normovolemic
		Humeral	7	NA	7	0	
Wong, 2015	Epinephrine	Tibial	7	508.6 ± 68.9	5	2	Normovolemic
		IV	7	680.8 ± 111.4	4	3	
Johnson, 2015	Epinephrine	IV	7	NA	4	3	Normovolemic
		Humeral	7	NA	7	0	
Beaumont, 2016	Epinephrine	Tibial	7	508 ± 154	5	2	Normovolemic
		IV	7	680 ± 223	4	3	
		Humeral	7	465 ± 218	7	0	
Long PR, 2018	Epinephrine	IV	7	NA	4	3	Normovolemic
		Humeral	7	NA	7	0	
Hoernchen, 1992	Epinephrine	IV	8	294 ± 168	8	0	Normovolemic
		Endotracheal	8	282 ± 108	8	0	
Burgert, 2017	Vasopressin	IV	11	554.50 ± 213.96	8	3	Normovolemic
		Humeral	7	621.2 ± 204.21	5	2	
Johnson, 2015	Vasopressin	Tibial	7	399 ± 110	7	0	Normovolemic
		IV	7	541 ± 226	7	0	
Burgert, 2019	Epinephrine	Tibial	7	476 ± 105.96	4	3	Hypovolemic
		IV	7	465 ± 119.46	4	3	
		Sternal	7	354 ± 69.51	3	4	
		Humeral	7	756 ± 303.72	4	3	
		Endotracheal	7	376 ± 84.36	2	5	

Beaumont, 2022	Epinephrine	IV	7	NA	4	3	Hypovolemic
		Humeral	7	NA	3	4	
Long PR, 2018	Epinephrine	IV	7	NA	4	3	Hypovolemic
		Humeral	7	NA	7	0	
Hampton, 2016	Amiodarone	Tibial	7	300 ± 67	6	1	Hypovolemic
		IV	7	184 ± 73	5	2	
Smith, 2016	Amiodarone	Sternal	7	287.5 ± 149.8	5	2	Hypovolemic
		IV	7	184 ± 132.2	5	2	

Supplementary Table 6: Meta-analysis for Continuous Outcomes

Outcome	Drug	Route/ Site of Administration	Status	Number of Studies	Number of Swine	Pooled Mean Difference (95% CI)	p-value
C_{max}	Epinephrine	IV vs IO	Normovolemic	6	76	108.69 (-572.95, 790.35)	0.6988
		IV vs Endotracheal		1	16	515 (415.90, 614.10)	<0.001
C_{max}	Amiodarone	IV vs IO	Normovolemic	2	28	-0.02 (-2.35, 2.32)	0.9403
C_{max}	Epinephrine	IV vs IO	Hypovolemic	4	56	382.80 (258.53, 507.07)	0.0022
		IV vs Endotracheal		1	14	425 (111.30, 738.70)	0.0079
C_{max}	Amiodarone	IV vs IO	Hypovolemic	2	28	17500.44 (-41340.8, 76341.7)	0.1647
T_{max}	Epinephrine	IV vs IO	Normovolemic	6	76	-21.01 (-77.63, 35.61)	0.3839
		IV vs Endotracheal		1	16	-82 (-96.42, -67.58)	<0.001
T_{max}	Amiodarone	IV vs IO	Normovolemic	2	28	-70.94 (-807.90, 666.02)	0.4363
T_{max}	Vasopressin	IV vs IO	Normovolemic	2	32	-0.698 (-1.70, 0.30)	0.0716
T_{max}	Epinephrine	IV vs IO	Hypovolemic	4	56	382.80 (258.53, 507.07)	0.0022

		IV vs Endotracheal		1	14	34 (-7.63, 75.63)	0.1094
T_{max}	Amiodarone	IV vs IO	Hypovolemic	2	28	-32.76 (-112.59, 47.08)	0.1206
Time to ROSC	Epinephrine	IV vs IO	Normovolemic	3	42	177.52 (134.40, 220.64)	0.0032
		IV vs Endotracheal		2	32	33.08 (-454.71, 520.87)	0.5472
	Vasopressin	IV vs IO	Normovolemic	2	32	40.27 (-1285.20, 1365.74)	0.7654
	Epinephrine	IV vs IO	Hypovolemic	3	42	-40.23 (-526.78, 446.31)	0.7560
		IV vs Endotracheal		1	14	89 (-19.34, 197.34)	0.1074
	Amiodarone	IV vs IO	Hypovolemic	2	28	-113.53 (-176.75, -50.31)	0.0279

Supplementary Table 7: Meta-analysis for Dichotomous Outcomes

Outcome	Drug	Route/ Site of Administration	Status	Number of Studies	Number of Swine	OR (95% CI)	p-value
ROSC achieved (OR)	Epinephrine	IV vs IO	Normovolemic	6	84	0.217 (0.07, 0.62)	0.0134
	Vasopressin	IV vs IO	Normovolemic	2	32	1.066 (0.129, 8.793)	0.9521
	Epinephrine	IV vs IO	Hypovolemic	5	70	1.015 (0.305, 3.374)	0.9744
	Amiodarone	IV vs IO	Hypovolemic	2	28	0.687 (2.79, 168.96)	0.5456

Supplementary Table 8: Meta-regression Table for Pharmacokinetic Outcomes

Outcome	Drugs	Comparison	Number of Studies	β	SE	p-value	95%CI
C_{max}	Epinephrine	IV vs IO	9	-231.87	266.95	0.4054	-826.68, 362.94
		IV vs Endotracheal	2	478.90	239.92	0.0739	-55.67, 1013.48
		Hypovolemic vs Normovolemic	12	-180.71	211.95	0.4138	-652.96, 291.54
		IV vs IO (Hypovolemic vs Normovolemic)	10	-363.62	524.57	0.5078	-1573.28, 846.04
C_{max}	Amiodarone	Hypovolemic vs Normovolemic	4	-17500.46	5426.62	0.0842	-40849.33, 5848.42
T_{max}	Epinephrine	IV vs IO	9	11.62	38.67	0.7699	-74.54, 97.79
		IV vs Endotracheal	2	-28.59	35.21	0.4357	-107.06, 49.87
		Hypovolemic vs Normovolemic	12	-26.98	28.16	0.3606	-89.71, 35.76
		IV vs IO (Hypovolemic vs Normovolemic)	10	107.43	73.74	0.1833	-62.62, 277.47
T_{max}	Amiodarone	Hypovolemic vs Normovolemic	4	-38.57	58.44	0.5771	-290.00, 212.87

Supplementary Table 9: Meta-regression Table for Pharmacodynamic Outcomes

Outcome	Drug	Moderator (Comparison)	Number of Studies	β (Estimated)	SE	p-value	95% CI
Time to ROSC	Epinephrine	IV vs Endotracheal	3	63.37	79.94	0.4540	-125.67, 252.41
		IV vs IO	6	13.68	97.88	0.8928	-217.76, 245.12
		Hypovolemic vs Normovolemic	9	109.39	79.20	0.2097	-77.89, 296.68
		IV vs IO (Hypovolemic vs Normovolemic)	6	238.51	169.01	0.2173	-195.95, 672.97
ROSC achieved (OR)	Epinephrine	IV vs Endotracheal	2	0.92	0.92	0.3436	-1.13, 2.98
		IV vs IO	11	-1.60	1.00	0.1382	-3.80, 0.60
		Hypovolemic vs Normovolemic	13	-1.64	0.57	0.0150	-2.89, - 0.38
		IV vs IO (Hypovolemic vs Normovolemic)	11	-0.34	1.92	0.8630	-4.68, 3.997

Supplementary Table 10: Meta-analysis for continuous outcomes (Subgroup Analysis)

Outcome	Drug	Route/ Site of Administration	Status	Number of Studies	Number of Swine	Pooled Mean Difference (95% CI)	p-value
C_{max}	Epinephrine	Tibial vs IV	Normovolemic	3	38	-348.16 (-887.55, 191.22)	0.1089
		IV vs Humeral		2	28	-157.19 (-231.92, -82.45)	0.0238
		IV vs Endotracheal		1	16	515 (415.90, 614.10)	<0.001
		Tibial vs Sternal		1	10	-3553 (-9455.86, 2349.86)	0.2381
C_{max}	Amiodarone	Tibial vs IV	Normovolemic	1	14	-0.17 (-0.35, 0.01)	0.0681
		Tibial vs Sternal		1	14	-0.37 (-0.55, -0.19)	<0.001
C_{max}	Vasopressin	Tibial vs IV	Normovolemic	1	14	-32.09 (-54.92, -9.25)	0.0059
		IV vs Humeral		1	18	-9.90 (-33.84, 14.04)	0.4177
C_{max}	Epinephrine	Tibial vs IV	Hypovolemic	1	14	-444 (-574.84, -313.16)	<0.001
		IV vs Humeral		2	28	396 (395.99, 396)	<0.001
		IV vs Endotracheal		1	14	425 (111.30, 738.70)	0.0079
		Tibial vs Sternal		1	14	-200 (-321.89, -78.11)	0.0013

C_{max}	Amiodarone	Sternal vs IV	Hypovolemic	1	14	28540.2 (-91272.08, 148352.5)	0.6406
		Tibial vs IV		1	14	-17966.23 (-30017.3, -5915.15)	0.0035
T_{max}	Epinephrine	Tibial vs IV	Normovolemic	3	38	69.24 (61.16, 77.31)	< 0.001
		IV vs Humeral		2	28	30.00 (29.99, 30.00)	< 0.001
		IV vs Endotracheal		1	16	-82 (-96.42, -67.58)	< 0.001
		Tibial vs Sternal		1	10	96 (57.46, 134.54)	< 0.001
T_{max}	Amiodarone	Tibial vs IV	Normovolemic	1	14	129 (104.88, 153.12)	< 0.001
		Tibial vs Sternal		1	14	116 (91.88, 140.12)	< 0.001
T_{max}	Vasopressin	Tibial vs IV	Normovolemic	1	14	0.7 (-0.33, 1.73)	0.1825
		IV vs Humeral		1	18	3.13 (-46.79, 53.05)	0.9022
T_{max}	Epinephrine	Tibial vs IV	Hypovolemic	1	14	69 (50.77, 87.23)	< 0.001
		IV vs Humeral		2	28	1 (4, 4)	0.00
		IV vs Endotracheal		1	14	34 (-7.63, 75.63)	0.1094
		Tibial vs Sternal		1	14	82 (64.19, 99.81)	< 0.001
T_{max}	Amiodarone	Sternal vs IV	Hypovolemic	1	14	-1.55 (-2.84, -0.25)	0.0189
		Tibial vs IV		1	14	-0.97 (-2.18, 0.24)	0.1157

Time to ROSC	Epinephrine	IV vs Endotracheal	Normovolemic	2	30	33.08 (-454.71, 520.87)	0.5472
		Tibial vs IV		2	28	-172.16 (-173.16, -171.17)	< 0.001
		IV vs humeral		1	14	215 (-16.02, 446.02)	0.0681
	Vasopressin	IV vs humeral	Normovolemic	1	18	-66.70 (-263.86, 130.46)	0.5073
		Tibial vs IV		1	14	-142 (-328.20, 44.20)	0.13499
	Epinephrine	IV vs Endotracheal	Hypovolemic	1	14	89 (-19.34, 197.34)	0.1074
		Tibial vs IV		1	14	11 (-107.29, 129.29)	0.8554
		IV vs Humeral		1	14	-291 (-532.77, -49.23)	0.0183
		Tibial vs Sternal		1	14	122 (28.12, 215.88)	0.0109
	Amiodarone	Sternal vs IV	Hypovolemic	1	14	103.5 (-44.50, 251.50)	0.1705
		Tibial vs IV		1	14	116 (42.60, 189.40)	0.0019

Supplementary Table 11: Meta-analysis for Dichotomous Outcomes (Subgroup Analysis)

Outcome	Drug	Route/ Site of Administration	Status	Number of Studies	Number of Swine	OR (95% CI)	p-value
ROSC achieved (OR)	Epinephrine	Tibial vs IV	Normovolemic	2	28	1.875 (1.9, 1.9)	0.00
		IV vs Humeral		4	56	0.086 (0.086, 0.086)	0.00
		IV vs Endotracheal		1	16	1.00 (0.018, 56.46)	1.00
	Vasopressin	IV vs Humeral	Normovolemic	1	18	1.066 (0.129, 8.793)	0.9521
		Tibial vs IV		1	14	1.00 (0.017, 57.31)	1.00
	Epinephrine	IV vs Humeral	Hypovolemic	3	42	0.811 (0.029, 22.23)	0.811
		IV vs Endotracheal		1	14	3.33 (0.36, 30.70)	0.2879
		Tibial vs IV		1	14	1.00 (0.12, 8.31)	1.00
		Tibial vs Sternal		1	14	1.78 (0.21, 14.77)	0.5942
	Amiodarone	Tibial vs IV	Hypovolemic	1	14	2.40 (0.16, 34.93)	0.5217
		Sternal vs IV		1	14	1.00 (0.098, 10.17)	1.00

Supplementary Table 12: Metaregression Table for Pharmacokinetic Outcomes (Subgroup Analysis)

Outcome	Drugs	Comparison	Number of Studies	B	SE	p-value	95%CI
C_{max}	Epinephrine	Tibial vs IV	4	-871.45	305.79	0.0215	-1576.61, -166.30
		IV vs Endotracheal	2	478.46	237.81	0.0790	-69.93, 1026.85
		IV vs Humeral	4	-340.88	288.52	0.2713	-1006.20, 324.45
		Tibial vs Sternal	2	-697.15	392.07	0.1133	-1601.26, 206.97
		Hypovolemic vs Normovolemic	6	-224.87	331.05	0.5124	-962.49, 512.74
		IV vs Humeral (Hypovolemic vs Normovolemic)	2	-643.19	266.41	0.0732	-1382.88, 96.50
		Tibial vs IV (Hypovolemic vs Normovolemic)	4	5.84	276.42	0.9842	-761.63, 773.30
		Tibial vs Sternal (Hypovolemic vs Normovolemic)	2	-3443.00	4372.64	0.4751	-15583.39, 8697.38
C_{max}	Amiodarone	Tibial vs IV	2	-36471.26	62920.05	0.6656	-835946.2, 763003.7

		Hypovolemic vs Normovolemic	4	17500.17	5423.02	0.0841	-5833.19, 40833.54
T_{max}	Epinephrine	Tibial vs IV	4	106.14	26.88	0.0042	44.15, 168.12
		IV vs Endotracheal	2	-36.09	21.42	0.1305	-85.48, 13.30
		IV vs Humeral	4	52.98	25.70	0.0732	-6.30, 112.25
		Tibial vs Sternal	2	123.95	30.28	0.0035	54.13, 193.77
		Hypovolemic vs Normovolemic	6	-0.86	30.49	0.9780	-68.80, 67.07
		IV vs Humeral (Hypovolemic vs Normovolemic)	2	142.00	3.51	<0.001	132.24, 151.76
		Tibial vs IV (Hypovolemic vs Normovolemic)	4	116.24	3.79	< 0.001	105.70, 126.77
		Tibial vs Sternal (Hypovolemic vs Normovolemic)	2	130.00	4.56	< 0.001	117.34, 142.65
T_{max}	Amiodarone	Tibial vs IV	2	39.04	89.14	0.7372	-1093.64, 1171.72
		Hypovolemic vs Normovolemic	4	89.74	9.06	0.0100	50.76, 128.72

Supplementary Table 13: Meta-regression Table for Pharmacodynamic Outcomes (Subgroup Analysis)

Outcome	Drug	Moderator (Comparison)	Number of Studies	β (Estimated)	SE	p-value	95% CI
Time to ROSC	Epinephrine	IV vs Endotracheal	3	64.07	90.94	0.5125	-169.70, 297.84
		Tibial vs IV	3	-170.68	125.72	0.2326	-493.86, 152.49
		IV vs Humeral	2	-96.29	158.65	0.5704	-504.11, 311.54
		Hypovolemic vs Normovolemic	9	-33.71	111.25	0.7707	-296.78, 229.36
		IV vs Humeral (Hypovolemic vs Normovolemic)	2	561.92	83.21	0.0212	203.90, 919.94
		Tibial vs IV (Hypovolemic vs Normovolemic)	3	-127.24	49.06	0.1221	-338.35, 83.87
ROSC achieved (OR)	Epinephrine	IV vs Endotracheal	2	0.92	0.90	0.3313	-1.11, 2.96
		Tibial vs IV	3	-0.52	1.07	0.6403	-2.94, 1.91
		IV vs Humeral	8	-2.08	1.02	0.0719	-4.38, 0.23
		Hypovolemic vs Normovolemic	13	-1.04	0.73	0.1798	-2.65, 0.56
		IV vs Humeral (Hypovolemic vs Normovolemic)	8	-1.04	1.66	0.5521	-5.10, 3.01
		Tibial vs IV (Hypovolemic vs Normovolemic)	3	1.83	1.74	0.3326	-2.42, 6.09
